# Comparative efficiency research (COMER): meta-analysis of cost-effectiveness studies

**DOI:** 10.1186/1471-2288-14-139

**Published:** 2014-12-22

**Authors:** Carlos Crespo, Antonio Monleon, Walter Díaz, Martín Ríos

**Affiliations:** Statistical Department, Facultat de Biologia, University of Barcelona, Avda Diagonal 645, 08028 Barcelona, Spain; Health Economics Outcomes Research and Pricing Department, Boehringer Ingelheim, Spain, Barcelona, Spain; Facultad de Ciencias Económicas, Universidad de Antioquia, Calle 67 No. 53-108, Medellín, Columbia

**Keywords:** Cost-effectiveness analysis, Meta-analysis, Incremental net benefit, Copula distribution

## Abstract

**Background:**

The aim of this study was to create a new meta-analysis method for cost-effectiveness studies using comparative efficiency research (COMER).

**Methods:**

We built a new score named total incremental net benefit (TINB), with inverse variance weighting of incremental net benefits (INB). This permits determination of whether an alternative is cost-effective, given a specific threshold (TINB > 0 test). Before validation of the model, the structure of dependence between costs and quality-adjusted life years (QoL) was analysed using copula distributions. The goodness-of-fit of a Spanish prospective observational study (n = 498) was analysed using the Independent, Gaussian, T, Gumbel, Clayton, Frank and Placket copulas. Validation was carried out by simulating a copula distribution with log-normal distribution for costs and gamma distribution for disutilities. Hypothetical cohorts were created by varying the sample size (n: 15–500) and assuming three scenarios (1-cost-effective; 2-non-cost-effective; 3-dominant). The COMER result was compared to the theoretical result according to the incremental cost-effectiveness ratio (ICER) and the INB, assuming a margin of error of 2,000 and 500 monetary units, respectively.

**Results:**

The Frank copula with positive dependence (−0.4279) showed a goodness-of-fit sufficient to represent costs and QoL (p-values 0.524 and 0.808). The theoretical INB was within the 95% confidence interval of the TINB, based on 15 individuals with a probability > 80% for scenarios 1 and 2, and > 90% for scenario 3. The TINB > 0 test with 15 individuals showed p-values of 0.0105 (SD: 0.0411) for scenario 1, 0.613 (SD: 0.265) for scenario 2 and < 0.0001 for scenario 3.

**Conclusions:**

COMER is a valid tool for combining cost-effectiveness studies and may be of use to health decision makers.

**Electronic supplementary material:**

The online version of this article (doi:10.1186/1471-2288-14-139) contains supplementary material, which is available to authorized users.

## Background

Economic evaluation of health technologies (EEHT) has become a first-order health policy decision making tool at the European level. EEHT allows both the economic and clinical value of technologies (drugs, devices, health programs, etc.) to be evaluated [1] and, when used for the allocation of health resources, allows decision-makers to make informed decisions on specific problems by combining the probabilities of all possible outcomes and the health benefits assigned to each of them [2, 3].

In the last decade, there has been a rise in EEHT, as reflected by the large amount of cost-effectiveness studies [4, 5] and the interest in the technological advances generated by their results. Although cost-effectiveness analyses (CEA) have improved due to the development of methods of synthesizing evidence, systematic revisions and direct, indirect and mixed meta-analyses [6, 7], there is still no consensual method for carrying out meta-analyses of CEA. Proposed methods range from mere qualitative review of CEA [8, 9] to the categorization of CEA according to whether the costs are higher, the same or lower and whether the effects are better, the same or poorer, in order to assess how many CEA there are in each category [10]. We suggest that the method for meta-analyses of CEA proposed here, which is known as COMparative Efficiency Research (COMER), and which groups together studies on a specific topic, may represent reality more closely and allow more evidence-based decision making. As for any meta-analysis, the validity of the COMER method will depend on systematic review of the types of studies supporting the data, the populations assessed and the variability or levels of consistency between centres and/or studies.

Although meta-analyses usually use aggregate study data, recent research has shown that the use of individual-level data or individual- and aggregate-level data, allows for a better estimate of variability [11, 12]. Therefore, the COMER method should be able to use both individual and aggregate data, although it remains difficult to obtain original data from clinical studies for use by researchers other than the original authors [13].

The aim of this study was to create a method of CEA meta-analysis based on either individual or aggregate data through the generation of a multivariate distribution function which allows the dependence between costs and effects to be modelled. This mathematical dependence is called copula.

## Methods

Cost-effectiveness studies compare the efficiency of therapies evaluated using the incremental cost-effectiveness ratio (ICER), where the numerator is the difference between the estimated cost of the new treatment and that of the reference treatment and the denominator estimates the effectiveness gained by the new treatment compared with the reference treatment [1].
1

where 1 and 2 correspond to the new treatment and the reference treatment, respectively.

Thus, when the therapy being assessed is more expensive than the reference treatment, and is also less effective, the new treatment is dominated. When the treatment is more effective than the reference treatment, and also less expensive, the new treatment is dominant over the reference treatment. In the remaining cases, the ICER must be compared with the willingness to pay (k) to assess whether the new treatment is cost-effective (ICER < = k) or not cost-effective (ICER > k) [1, 2]. The choice of the threshold of efficiency remains an unresolved issue in many health systems. Theoretically, the threshold of efficiency should be related to the value that society accords to a health outcome in, for example, life years gained, depending on the available resources [1, 2].

One limitation of the ICER is that, as it is a ratio, the expected value of the ICER represents the difference in the expected cost divided by the difference in the expected effectiveness. Therefore, the main difficulty in making inferences from cost-effectiveness studies is that the random variable obtained is not necessarily normal or symmetric [14]. However, even when cost and effectiveness data are distributed normally, there is no guarantee that the ratio between them will also behave normally and, ultimately, it is not possible to calculate the confidence intervals (CI). Thus, there is a need to use alternative methods, such as bootstrapping or incremental net benefits (INB) for this purpose [15].

Therefore, the COMER methodology, both for individual and aggregate data, consists of estimating the total incremental net benefit (TINB) according to the INB of the studies included weighted by the inverse of the variance of each INB.
2

Where s is the number of the study and 1 and 2 the new treatment and reference treatment, respectively:

and
3

and the variance is:
4

According to the central limit theorem, the INB is asymptotically normal [15] and the TINB is also asymptotically normal when the mean and variance is known, and the probability of the TINB being < 0 can be calculated. Therefore, the decision criterion will be that the new treatment is cost-effective for the established threshold if the probability is lower than 5% (Table 1).

Equivalently,


with

**Table 1:** **Summary of COMER outcomes**

Study	Alternative 1		Alternative 2		ICER	INB (variance)	INB <0 (%)*	Weight (%)
	Costs	Effects	Costs	Effects				
1	c_11_	e_11_	c_12_	e_12_	ICER_1_	INB_1_ (Var(INB_1_))	P(x_1_<0)	ω_1_
2	c_21_	e_21_	c_22_	e_22_	ICER_2_	INB_2_ (Var(INB_2_))	P(x_2_<0)	ω_2_
3	c_31_	e_31_	c_32_	e_32_	ICER_3_	INB_3_ (Var(INB_3_))	P(x_3_<0)	ω_3_
	Σ ω_s_^*^c_s1_	Σ ω_s_^*^e_s1_	Σ ω_s_^*^c_s2_	Σ ω_s_^*^e_s2_	ICER_S_	TINB =Σ ω_s_ *INB_s_, (Var(TINB))	P(TINB<0)	Σ ω_s_=1

ICER: Incremental Cost-effectiveness Ratio; INB: Incremental Net Benefit.

TINB: Total Incremental Net Benefit.

### Validation of the method

The costs and effects of individual patients were modelled for marginal distributions to illustrate the COMER method. The use of the copula distribution, which permits the joint distribution of costs and effects to be obtained, has been suggested [16], since the dependence between costs and effects does not have to be linear. In this way, a cost and effect dependence structure is obtained through the copula and their univariate behaviour.

The process carried out consisted of:

- Identifying the copula: the joint distribution that best fits the costs and effects.
- Creating a simulated cohort under the marginal and joint distribution for each alternative compared.
- COMER estimation.
- Method validation.

### Identification of marginal distributions and copula

Copulas are bivariate distributions that provide dependent structures for two statistical variables, with any type of univariate distribution [17–19]. In its construction, a C(u,v) copula is a multivariate distribution function defined from U and V random variables with uniform distribution in the interval [0,1], which verifies the following properties:

1. *C*(*u*, 0) = 0 = *C*(0, *v*)
2. *C*(*u*, 1) = *u and C*(1, *v*) = *v*
3. *For u*1, *u*2, *v*1, *v*2 *in* [0, 1] *so that u*1 ≤ *u*2 *and v*1 ≤ *v*2, *C*(*u*2, *v*2) − *C*(*u*2, *v*1) − *C*(*u*1, *v*2) + *C*(*u*1, *v*1) ≥ 0.

Since the relationship between random variables is not based on the distribution, but rather constructed from mathematical structures between random variables, dependence can be evaluated using non-parametric correlation statistics (Spearman’s ρ_s_ and Kendall’s τ), which are independent on marginal distributions.

Copula distributions are little used in health economics, and are mainly applied to specify the distribution of regression models with more than one dependent variable [20–23]. We used copulas to describe the dependence structure of cost-effect random variables, allowing random generation of a patient cohort under such a distribution in order to make different simulations. By applying copulas to EEHT, the joint distribution of each treatment can be simulated, since C_j_(c_ij_,e_ij_) is a copula for treatment j, where costs (c) and effects (e) are known for each patient i. In addition, Sklar’s theorem [24] shows that, given a copula (the joint distribution), the copula can be reconstructed through the marginal theoretical or empirical inverse distribution function, according to the specific case. However, recent studies show that there is no justification for the claim that a specific copula may be the most appropriate for the combination of costs and effects, not even when costs are broken down into direct and indirect costs, or if effects are represented by the following measurements: therapeutic success, life years, or quality-adjusted life years (QALY) [25].

To create a hypothetical population to test the validity of the COMER methodology, we used data from a Spanish prospective observational study of patients with allergic rhinitis (n = 498) with direct costs (c) and mean utilities from the SF-12v1 (e) [26]. The study served to obtain a copula structure which, by adding some theoretical marginal distributions, allowed the generation of a hypothetical population on which to apply the COMER methodology. The empirical copula was compared to the parametric estimate of the potential copula (Additional file 1) to evaluate the goodness of fit of the data with the theoretical copulas [27]. The copula bound to evaluate the goodness of fit was developed using the inversion of Kendall’s τ. The p-value of the test was calculated by simulation of size-100 bootstrap, due to the lack of an analytical construction.

The following copulas were evaluated [19, 25, 28]:

Independent copula: this can be generated automatically since, given U and V random variables, the joint distribution function is *C*^0^(*u*, *v*) = *u* * *v*. Thus, the association between this copula and some data indicates the stochastic independence of U and V; equivalently, the absence of structure.

Gaussian copula: this is defined as *G*_∅_(*u*, *v*) = *N*_*ρ*_(Φ^− 1^(*u*), Φ^− 1^(*v*)) where *N*_*ρ*_(x, y) is the normal distribution function of parameters x: mean and y: the standard deviation, Φ^− 1^(*x*) is the marginal distribution function N(0,1) and ρ is Pearson’s correlation coefficient. The Gaussian copula is still a multivariate normal distribution.

T copula: this is derived from the Student’s t multivariate distribution and is defined as  where |ρ| < 1, θ > 0. This copula shows a similar structure to the Gaussian copula, but presents tail dependence, points (0,1) and (1,0). One of its qualities is that it includes the Gaussian copula when θ → ∞.

Gumbel copula: this allows a positive dependence structure to be modelled, region (1,1), and is defined as  where *θ* ∈ [1, ∞]. When *θ* = 1 it is equivalent to the independent copula and when θ → ∞ it behaves as a comonotonic copula (min(u,v)). Thus, the behaviour of the Gumbel copula is an interpolation between the independent copula and the copula of perfect positive dependence.

Clayton copula: this is defined as  where *θ* > 0. Like the Gumbel copula, this copula is an interpolation but lies between the independent copula and the perfect negative dependence (point (0,0)).

Frank Copula: this has the quality of showing symmetric dependencies and not showing dependence at points (0,0) and (1,1). It is defined as  where θ ∈ ℝ \{0}.

Plackett copula: this is defined as  where θ ≥ 0. This structure shows both positive and negative maximum dependence in function of parameter *θ*.

### Creation of cohorts

Once the joint distribution was known, some theoretical marginal distributions were associated. To simulate costs, a lognormal distribution was associated, and to simulate life quality, a gamma distribution was associated (disutilities), with respect to a baseline quality of life (0.9 utility) [14] (Table 2). For the two alternatives, the same copula and the same randomization (bivariate uniforms [0,1]) were used. This ensured covariance and correlation between the values generated for the costs and effects. Different random samples per cohort were created with sample sizes between 15 and 500 individuals for each alternative. Individuals in the simulated cohort were randomly assigned to the studies. To ensure that there was a minimum variability per study, the random assignation was conditioned to ensure that, for each study, there were at least 3 individuals.

**Table 2:** **Simulated scenarios**

**Scenario 1:** **Cost**-**effective**		
	**CONTROL**	**ACTIVE**
Cost	Lognormal (6.214608, 0.75)	Lognormal (6.907755, 0.75)
Effect	0.9-Gamma (6.25, 0.024)	0.9-Gamma (2, 0.05)
ICER	13,247.85 = (1,324.78 - 662.39)/(0.8-0.75)	
INB*	837.61	
**Scenario 2:** **Non**-**cost**-**effective**		
	**CONTROL**	**ACTIVE**
Cost	Lognormal (6.214608, 0.75)	Lognormal(6.214608, 0.75)
Effect	0.9-Gamma (6.25, 0.024)	0.9-Gamma (4.5, 0.03)
ICER	44,159.49 = (1,324.78 - 662.39)/(0.765-0.75)	
INB*	−212.39	
**Scenario 3:** **Dominant**		
	**CONTROL**	**ACTIVE**
Cost	Lognormal (6.907755, 0.75)	Lognormal (6.214608, 0.75)
Effect	0.9-Gamma (6.25, 0.024)	0.9-Gamma (2, 0.05)
ICER	−13,247.85 = (662.39 – 1,324.78)/(0.8-0.75)	
INB*	2,162.39	

ICER: Incremental Cost-effectiveness Ratio; INB: Incremental Net Benefit.

*k=30,000.

The COMER methodology was applied to the simulated data for each alternative. For each alternative the mean costs, mean effectiveness, differential variance in costs, differential variance in effects and covariance between the differences in costs and effects, and the INB were estimated, setting an efficiency threshold (k = 30,000 monetary units per QALY gained). For example, of a sample of 15 individuals, the seven first could be assigned to study 1, the following five to study 2, and the remaining three to study 3, and the COMER methodology applied (Additional file 2).

For each sample size generated, 500 replications were made, entailing 25,000 meta-analyses for each scenario, thus allowing the number of the times the methodology agreed with reality to be validated. A 2,000-monetary-unit/QALY-tolerance was assumed to calculate the ICER, and a 500-monetary-unit-tolerance was assumed for the TINB. Additionally, we estimated the minimum sample size required to obtain an adjusted estimate with a probability >70% and when simulations converged to the original Kendall’s τ.

The following are the scenarios evaluated by modifying the marginal distribution parameters (Table 2):

1. The second alternative is cost-effective.
2. The second alternative is more costly and more effective, although the ICER is above the willingness to pay threshold.
3. The second alternative is dominant.

All the possibilities are covered by these scenarios since the remaining potential outcomes derived from an EEHT which have not explicitly been evaluated are obtained assuming that the current alternative is the second alternative instead of the first.

The analysis was made using the R 3.0.1 statistical package (Additional file 3). In addition, the COMER method is included as an Excel file in the supplementary materials (Additional file 4).

## Results

Comparison of the copulas used with the real costs and effects data showed p-values between 0.075 and 0.191 for the independent distribution, 0.443 and 0.769 for the Gaussian copula, 0.309 and 0.633 for the T copula, 0.004 and 0.034 for the Gumbel copula, 0.242 and 0.526 for the Clayton copula, 0.524 and 0.808 for the Frank copula and 0.531 and 0.808 for the Plackett copula (Table 3).

**Figure 1:**
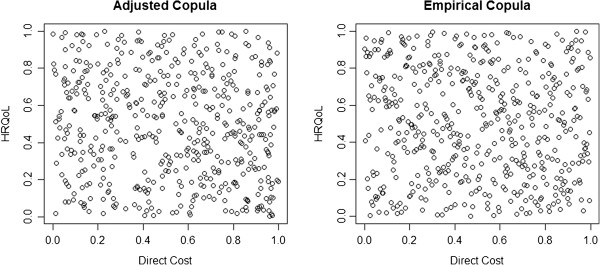
**Adjusted Copula (Frank Copula) vs. Empirical Copula.**

Therefore, both the Frank and Plackett copulas were candidates to be the copula for the distribution of the simulated cohort. We opted for the Frank copula, which showed a value of −0.4279 (p-value: 0.128), indicating a positive dependence between costs and effects (Figure 1).

**Table 3:** **Goodness**-**of**-**fit of p values (100 times)**

	Minimum	1st quartile	Median	Mean	3rd quartile	Maximum
Independent	0.075	0.105	0.121	0.119	0.129	0.191
Gaussian	0.443	0.558	0.61	0.599	0.637	0.769
T	0.309	0.457	0.49	0.489	0.532	0.633
Gumbel	0.004	0.011	0.014	0.015	0.018	0.034
Clayton	0.242	0.355	0.398	0.395	0.435	0.526
Frank	0.524	0.663	0.695	0.696	0.738	0.808
Plackett	0.531	0.668	0.71	0.703	0.739	0.808

Evaluation of the convergence from the empirical to the theoretical Kendall’s τ showed a range between −0.505 and 0.543 for 15 simulated individuals (mean: 0.029; SD: 0.192), between −0.135 and 0.228 for 115 individuals (mean: 0.046; SD: 0.062) and between −0.059 and 0.130 for 415 individuals (mean: 0.049; SD: 0.029).

**Figure 2:**
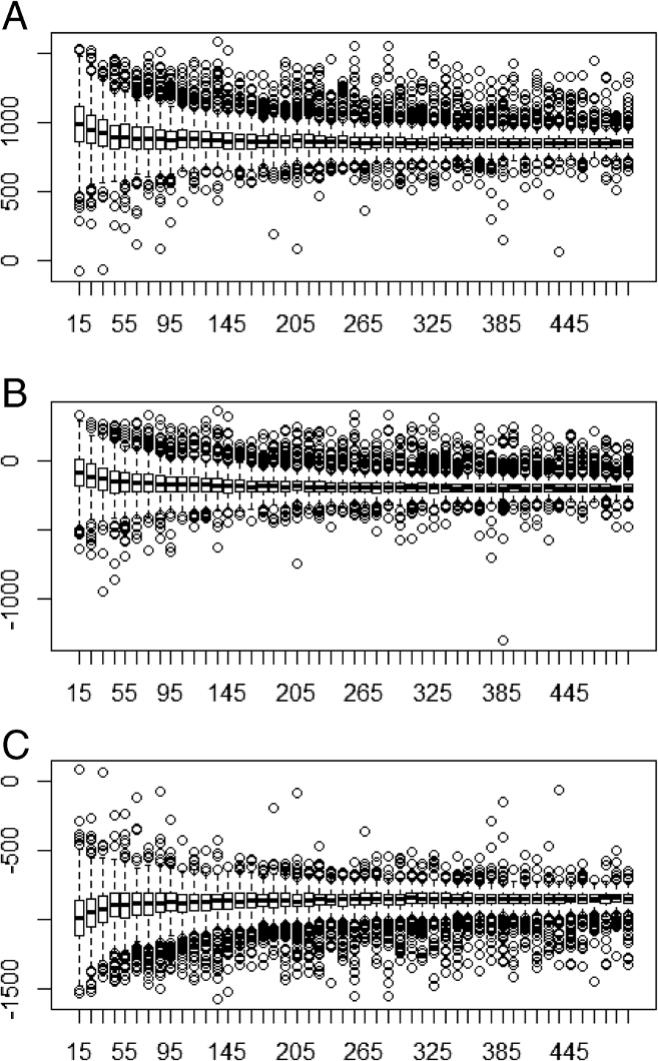
**Boxplot of TINB convergence. A**: Cost-effective scenario; **B**: Non-cost-effective scenario; **C**: Dominant scenario.

1. **Cost-effective scenario**
  In the cost-effectiveness scenario it was observed that, starting from a sample size of 75 individuals per cohort, both the ICER and the INB were within the tolerance. It was also observed that, when the number of simulated individuals was increased, the adjustment increased with probabilities > 90%.
  Comparison of the INB with the individual data and the TINB showed a minimum of 175, 115 and 75 individuals were necessary to obtain a probability greater than 70%, when tolerances of 6%, 9% and 12%, respectively, were assumed. However, the INB was within the CI of the TINB with a probability > 85% for samples of > 15 individuals and > 90% for samples > 25 individuals. The same happened when the CI of the TINB for each meta-analysis was compared with the theoretical value of the INB. (+80% for 15+ individuals and +90% for 25+ individuals) (Figure 2A).
  With respect to the probability of alternative 2 being cost-effective, for the sample size of 15 individuals, the p-value was 0.0105 (SD: 0.0411), for 115 individuals it was 0.015 (SD: 0.0214) and for 415 individuals it was 0.0165 (SD: 0.0128). Thus, for the meta-analyses obtained, alternative 2 was cost-effective.
2. **Non-cost-effective scenario**
  The ICER reached values > 70% within the tolerance, from 400 individuals upwards. Because the denominator of the ICER had a mean difference of 0.01 QALYs, the ICER showed values between 19,840 and 103,436 for n: 15. This meant that, in 7% of the simulations with n: 15 and in 1% of those with n:35,the ICER was inferior to the willingness-to-pay, and was thus cost-effective. However, the probability of the INB being within the tolerance (500 monetary units) was > 95% for any sample size.
  Comparison of the INB with the individual data with respect to the 95% CI of the TINB showed a probability of 85% for samples > 15 individuals. The same occurred when the 95% CI of the TINB for each meta-analysis was compared with the theoretical value of the INB (+80% for 15+ individuals and +90% for 25+ individuals) (Figure 2B). The mean difference between the mean TINB and the theoretical value showed a range of 15 to 121 monetary units.
  With respect to the probability of alternative 2 being cost-effective, for the sample size of 15 individuals, the p-value was 0.613 (SD: 0.265), for 115 individuals it was 0.709 (SD: 0.123) and for 415 individuals it was 0.729 (SD: 0.072). Thus, for the meta-analyses obtained, the outcome showed that alternative 2 was not cost-effective.
3. **Dominant scenario**
  The ICER reached values > 70% within the tolerance, from 35 individuals upwards, while the INB reached these values from 15 individuals upwards. Furthermore, when comparing the INB with the individual data and the theoretical INB with a 6% tolerance, a probability > 70% was obtained from 35 individuals upwards.
  When the INB of the individual data or the theoretical INB was within the CI, probabilities > 90% were observed for any sample size (Figure 2C). Regarding the probability of alternative 2 being cost-effective, scenario 3 showed p-values < 0.0001 for any sample size.

## Discussion

EEHT are a set of tools which aim to examine the short and long-term consequences of using health technologies on individuals and on society. Since there are numerous alternatives for allocating those resources, EEHT tries to make relevant information from the efficiency perspective available to health decision makers, understanding efficiency as the maximization of health gains obtained from the limited resources available. The application of COMER in decision-making may allow decision-makers to move from evidence-based medicine to economic evaluation based on evidence, understanding evidence within a comprehensive framework where both clinical and economic evidence are compatible. COMER may be applied both in studies where individual level data or aggregate level data are available, always provided the covariance matrix of the difference between costs and effects, the mean cost and the mean effects of each alternative are available. When there is access to individual data from clinical studies, these measurement can be obtained through bootstrapping [29–31], and in the case of economic evaluation models, they can be obtained from the Monte Carlo simulation [14]. The advantage of bootstrapping is that it is a non- parametric method which does not presuppose any distribution and is based on studying the sample as if it were the population, through resampling [30].

We believe that the fact that the simulated individuals were only assigned to 3 studies did not influence the results. Although many meta-analyses agglutinate more than 3 studies, the number of studies used was the minimum required to enable evaluation of the generalization of the results. In addition, in the assignation of individuals per study we forced the assignation of three or more individuals to avoid studies with minimum or null variability. Although, in reality it is unusual to find studies with 3 cases, analysis of very-small samples allows a small number of individuals to be assigned, thus generating greater heterogeneity between studies, as occurs in reality. However, the analysis with small simple sizes showed, as expected, greater divergence in the results obtained using the ICER and the TINB. Likewise, using two theoretical distributions such as the lognormal for costs and the gamma for disutilities is sufficient to validate the method. Furthermore, since approximation by copulas is independent of marginal distributions, and because typical theoretical distributions were selected to simulate the uncertainty of the costs and disutilities, we believe that the outcome is not biased by this decision. However, any interpretation of the COMER methodology must take into consideration the field in which it has been implemented and validated. In addition, the meta-analysis constructed has fixed effects (inverse variance weighting), which means it must be assumed that the studies are homogenous. This assumption does not necessarily occur in reality, and even though, to validate the method, different sample sizes and high levels of variance (e.g., a coefficient of variation for the costs >1) were used to generate greater heterogeneity in the study data, this should be taken into account when interpreting the results. Future studies should use the COMER methodology to develop a method to adjust the divergences in the homogeneity of the populations evaluated, similar to that carried out for meta-regressions.

A potential limitation to applying the COMER method is that health systems in different countries can generate different resource uses and, hence, different total costs. For this reason, the transfer of economic evaluations must be assessed and costs must be homogenized before using this method [32–34]. When using studies from different countries with potentially-similar cost structures, the currencies must be homogenized to US dollar equivalents and costs must be updated to the same year, as is done in comparisons made by the World Health Organization [35], the OECD [36] or the IMF [37].

Some authors suggest that a separate cost and effectiveness meta-analysis is sufficient to make an appropriate value judgment [38], while other suggest assessment of the results of the costs and marginal effects of CEA in order to evaluate the worth of a new treatment [10]. However, we believe that the COMER method has potential applications, even for teams with limited resources. Mere systematic review and meta-analysis, separate from economic evaluation studies, are not an EEHT per se, as they could lead decision makers to draw the wrong conclusion due to the impossibility of balancing global costs and effects and measuring additional value. EEHT should be accompanied by a fuller meta-analysis such as the proposed COMER method, although a full, new EEHT might be necessary if sufficient information were not obtained by the COMER method.

## Conclusions

Systematic review of economic analyses requires methods to synthesize and interpret the results of multiple analyses. The COMER methodology is a valid option for the analysis of both individual and aggregate data and may allow decision-makers to better understand the added value of new therapeutic alternatives by synthesizing all available evidence. The COMER methodology opens the way to further research of this type of meta-analysis with two variables having a known dependence structure [16], both from a frequentist and from a Bayesian perspective [39].

## Electronic supplementary material

Additional file 1: **Copula distributions and associated correlation.** (DOC 276 KB)

Additional file 2: **Example of the COMER with a sample size of 15 (scenario cost-effective).** (DOC 66 KB)

Additional file 3: **Script COMER function in R.** (DOC 31 KB)

Additional file 4: **Comparative efficiency research (COMER) template.** (XLSX 17 KB)

## Abbreviations

CEA: Cost-effectiveness analyses
CI: Confidence interval
COMER: Comparative efficiency research
EEHT: Economic evaluation of health technologies
ICER: Incremental cost-effectiveness ratio
INB: Incremental net benefits
QALY: Quality adjusted life years
SD: Standard deviation
TINB: Total incremental net benefit.
