# Supplementary material for: Comparative efficiency research (COMER): meta-analysis of cost-effectiveness studies
Source: BMC Med Res Methodol. 2014 Dec 22;14:139. doi: 10.1186/1471-2288-14-139 (PMC4292992; doi:10.1186/1471-2288-14-139)
Supplement: Supplementary file 1 — Additional file 1: Copula distributions and associated correlation. (DOC 276 KB) [file 12874_2014_1149_MOESM1_ESM.doc]

Additional file 1: Copula distributions and associated correlation

|  | Copula | Sperman | Tau kendall |
| --- | --- | --- | --- |
| Independent | 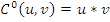 | ρ_s_=0 | 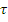 =0 |
| Gaussian | 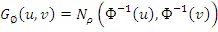 | -1≤ρ_s_≤1  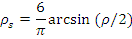 | -1≤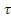 ≤1  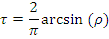 |
| T | 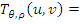  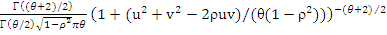 | 1≤ρ_s_≤1  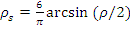 (n>30) | -1≤τ≤1  *𝜏=*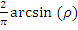 (n>30) |
| Gumbel | 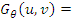  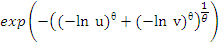 | -0.5238≤ρ_s_≤0  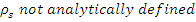 | -0.3613≤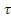 ≤0  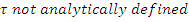 |
| Clayton | 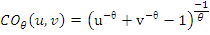 | 1≤ρ_s_≤1  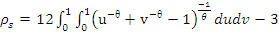 | -1≤τ≤1  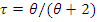 |
| Frank | 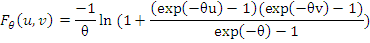 | 1≤ρ_s_≤1  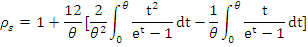 | -1 ≤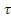 ≤1  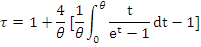 |
| Placket | 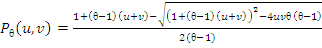 | 1≤ρ_s_≤1  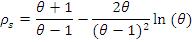 | -1 ≤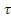 ≤1  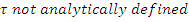 |
