# Supplementary material for: Comparative efficiency research (COMER): meta-analysis of cost-effectiveness studies
Source: BMC Med Res Methodol. 2014 Dec 22;14:139. doi: 10.1186/1471-2288-14-139 (PMC4292992; doi:10.1186/1471-2288-14-139)

Additional file 2: Example of the COMER with a sample size of 15 (SCENARIO COST-EFFECTIVE).

Random sample

| **Id** | **Study** | **Alternative 1** | | **Alternative 2** | |
| --- | --- | --- | --- | --- | --- |
|  |  | **Costs** | **Effects** | **Costs** | **Effects** |
| **1** | 1 | 328.41 | 0.667 | 656.81 | 0.701 |
| **2** | 1 | 911.44 | 0.797 | 1822.88 | 0.855 |
| **3** | 1 | 420.72 | 0.835 | 841.45 | 0.883 |
| **4** | 1 | 1220.80 | 0.787 | 2441.60 | 0.846 |
| **5** | 1 | 1609.44 | 0.643 | 3218.87 | 0.668 |
| **6** | 1 | 140.81 | 0.667 | 281.63 | 0.702 |
| **7** | 1 | 527.15 | 0.727 | 1054.30 | 0.780 |
| **8** | 2 | 1266.78 | 0.742 | 2533.55 | 0.798 |
| **9** | 2 | 550.91 | 0.557 | 1101.82 | 0.538 |
| **10** | 2 | 460.76 | 0.733 | 921.53 | 0.787 |
| **11** | 2 | 1809.68 | 0.731 | 3619.37 | 0.785 |
| **12** | 2 | 457.91 | 0.751 | 915.82 | 0.808 |
| **13** | 3 | 706.48 | 0.746 | 1412.96 | 0.803 |
| **14** | 3 | 573.59 | 0.789 | 1147.19 | 0.847 |
| **15** | 3 | 193.60 | 0.806 | 387.21 | 0.863 |

Summary statistics

| **Study** | **Differential variance in costs** | **Differential variance in effects** | **Covariance between the differences in costs and effects** |
| --- | --- | --- | --- |
| 1 | 28,2015.3 | 0.00017781 | -0.1547603 |
| 2 | 367,964.3 | 0.001122231 | 6.359636 |
| 3 | 70,848.94 | 1.437118e-06 | 0.1108376 |

Summary COMER

| **Study** | **Alternative 1** | | **Alternative 2** | | **ICER** | **INB**  **(variance)** | **INB <0**  **(%)*** | **Weight (%)** |
| --- | --- | --- | --- | --- | --- | --- | --- | --- |
|  | **Costs** | **Effects** | **Costs** | **Effects** |  |  |  |  |
| 1 | 736.97 | 0.732 | 1,473.93 | 0.776 | 16,749.09 | 599.53 (451,329.85) | 0.186086902 | 12.0% |
| 2 | 909.21 | 0.703 | 1,818.42 | 0.743 | 22,730.25 | 306.84 (996,393.65) | 0.379271448 | 5.4% |
| 3 | 491.23 | 0.781 | 982.45 | 0.838 | 8,617.89 | 1,229.87 (65,492.09) | 7.70709E-07 | 82.6% |
|  | 543.37 | 0.77 | 1,086.73 | 0.83 | 9,056.00 | 1,104.22 (54,088.22) | 1.02755E-06 |  |

ICER: Incremental Cost-effectiveness Ratio; INB: Incremental Net Benefit

Forest Plot


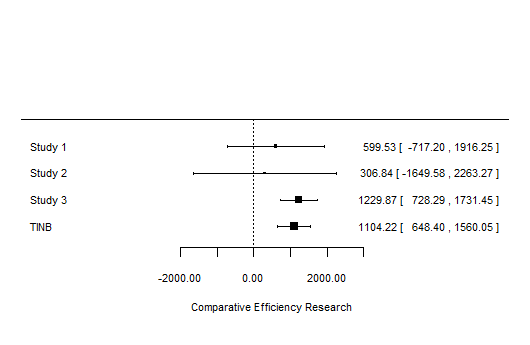

Supplement: Supplementary file 2 — Additional file 2: Example of the COMER with a sample size of 15 (scenario cost-effective). (DOC 66 KB) [file 12874_2014_1149_MOESM2_ESM.doc]
