# Supplementary material for: Comparative efficiency research (COMER): meta-analysis of cost-effectiveness studies
Source: BMC Med Res Methodol. 2014 Dec 22;14:139. doi: 10.1186/1471-2288-14-139 (PMC4292992; doi:10.1186/1471-2288-14-139)
Supplement: Supplementary file 3 — Additional file 3: Script COMER function in R. (DOC 31 KB) [file 12874_2014_1149_MOESM3_ESM.doc]

Additional file 3: Script COMER function in R

# COMER function

# myframe: study Id; cost1;efect1;cost2;efect2

# ngroup: # of studies

# k: threshold

# Forest function needs 'metafor' package

Library(‘metafor’)

COMER<-function(myframe, ngroup,k){

if (ngroup!= as.integer(ngroup))

return("# group must be integer")

if (ngroup < 2)

return("# group must be >= 2")

names(myframe)<-c("Id","C1","E1","C2","E2")

myframe$DifC<-myframe$C2-myframe$C1

myframe$DifE<-myframe$E2-myframe$E1

cat("Comparative Efficiency Research (COMER):\n")

cat("Study\tMean_Cost\t Mean_effect\tMean_Cost\tMean_effect\t\tINB\tVar(INB)\n")

SumInverseVar<-0

results<-array(dim=c(ngroup+1,12),dimnames=list(NULL,c("study","mC1","mC2","mE1","mE2","vDifC","vDifE","cDifCE","INB","vINB","p0INB","weight")))

for(s in 1:ngroup){

subframe<-subset(myframe,Id==s)

#means

mC1<-mean(subframe$C1,na.rm=TRUE)

mC2<-mean(subframe$C2,na.rm=TRUE)

mE1<-mean(subframe$E1,na.rm=TRUE)

mE2<-mean(subframe$E2,na.rm=TRUE)

#co/variance incrementals

vDifC<-var(subframe$DifC,na.rm=TRUE)

vDifE<-var(subframe$DifE,na.rm=TRUE)

cDifCE<-cov(subframe$DifC,subframe$DifE,method="pearson")

#INB

INB<-(mE2-mE1)*k-(mC2-mC1)

#Var(INB)

vINB<-vDifE*k^2+vDifC-2*k*cDifCE

SumInverseVar<-SumInverseVar+1/vINB

# % INB>0

p0INB<-pnorm(0,INB,sqrt(vINB))

#Save result study s

results[s,"study"]<-s

results[s,"mC1"]<-mC1

results[s,"mC2"]<-mC2

results[s,"mE1"]<-mE1

results[s,"mE2"]<-mE2

results[s,"vDifC"]<-vDifC

results[s,"vDifE"]<-vDifE

results[s,"cDifCE"]<-cDifCE

results[s,"INB"]<-INB

results[s,"vINB"]<-vINB

results[s,"p0INB"]<-p0INB

#print

p1<-paste(s,"\t",round(mC1,digit=2),"\t",round(mE1,digit=3))

p2<-paste("\t",round(mC2,digit=2),"\t",round(mE2,digit=3))

p3<-paste("\t",round(INB,digit=2),"\t",round(vINB,digit=2),"\n")

cat (paste(p1,p2,p3))

}

Label<-""

for(s in 1:ngroup){

results[s,"weight"]<-(1/results[s,"vINB"])/SumInverseVar

Label<-c(Label,paste("Study",s))

}

cat(paste(“Threshold”,k,"\n")

#INB Total

TINB<-results[-(ngroup+1),"INB"]%*%results[-(ngroup+1),"weight"]

#Var(INB Total)

vTINB<-1/SumInverseVar

# % INB Total>0

p0TINB<-pnorm(0,TINB,sqrt(vTINB))

#Save result study s

results[ngroup+1,"study"]<-0

results[ngroup+1,"mC1"]<-results[-(ngroup+1),"mC1"]%*%results[-(ngroup+1),"weight"]

results[ngroup+1,"mC2"]<-results[-(ngroup+1),"mC2"]%*%results[-(ngroup+1),"weight"]

results[ngroup+1,"mE1"]<-results[-(ngroup+1),"mE1"]%*%results[-(ngroup+1),"weight"]

results[ngroup+1,"mE2"]<-results[-(ngroup+1),"mE2"]%*%results[-(ngroup+1),"weight"]

results[ngroup+1,"vDifC"]<-results[-(ngroup+1),"vDifC"]%*%results[-(ngroup+1),"weight"]^2

results[ngroup+1,"vDifE"]<-results[-(ngroup+1),"vDifE"]%*%results[-(ngroup+1),"weight"]^2

results[ngroup+1,"cDifCE"]<-results[-(ngroup+1),"cDifCE"]%*%results[-(ngroup+1),"weight"]^2

results[ngroup+1,"INB"]<-TINB

results[ngroup+1,"vINB"]<-vTINB

results[ngroup+1,"p0INB"]<-p0TINB

results[ngroup+1,"weight"]<-1

#print

p1<-paste("\nTotal Increment Net Benefit (TINB):\t",round(TINB,digits=2),"\nVariance (TINB):\t",round(vTINB,digit=2))

p2<-paste("\n% Total INB<0:\t",round(p0TINB,digit=3),"\n")

cat (paste(p1,p2))

forest(x=results[,9],vi=results[,10],slab=c(Label[-1],"TINB"), xlab= "Comparative Efficiency Research",cex=.7)

return(results)

}
